# Supplementary material for: Identifying interactions in omics data for clinical biomarker discovery using symbolic regression
Source: Bioinformatics. 2022 Jun 22;38(15):3749–58. doi: 10.1093/bioinformatics/btac405 (PMC9344843; doi:10.1093/bioinformatics/btac405)
Supplement: btac405_Supplementary_Data [file btac405_supplementary_data.pdf]

Supplementary material

Benchmarks

In order to have a fair and comprehensive benchmark for the QLattice, we trained and evaluated other machine learning algorithms in combination with different feature selection techniques on all four datasets. We used the nested cross-validation scheme detailed in Cawley and Talbot [2010] for hyperparameter tuning and performance estimation. The algorithms tested are LASSO, Elastic Net, Random Forest and Gradient Boosting, and the feature selection techniques are LASSO, top N features based on Mutual Information, and top N features based on the F-test (where N is either ten or the number of features used by the QLattice models). All implementations are from scikit-learn Pedregosa *et al.* [2011]. Performance benchmarks on regression problems for the QLattice and other algorithms can be found in a previous work Wilstrup and Kasak [2021].

Upon running the Friedman test (implementation by SciPy Virtanen *et al.* [2020]) on the best performing models (QLattice, and Random Forest and Gradient Boosting in combination with feature selection based on Mutual Information and LASSO), we obtain a p-value of 0.62. Hence, we reject the hypothesis of one model being consistently better than the rest.

	All Features	MI top 10	F top 10	MI top 3	F top 3	LASSO Features
QLattice	0.929	-	-	-	-	-
LASSO	0.875	-	-	-	-	-
Elasticnet	0.657	-	-	-	-	-
Random Forest	0.943	0.953	0.955	0.934	0.951	0.982
Gradient Boosting	0.989	0.966	0.95	0.942	0.937	0.973

Table 1. Benchmarks for Alzheimer's Disase dataset

	All Features	MI top 10	F top 10	MI top 3	F top 3	LASSO Features
QLattice	0.957	-	-	-	-	-
LASSO	0.963	-	-	-	-	-
Elasticnet	0.841	-	-	-	-	-
Random Forest	0.957	0.966	0.967	0.954	0.957	0.98
Gradient Boosting	0.971	0.969	0.969	0.945	0.95	0.975

Table 2. Benchmarks for Insuline Response dataset

	All Features	MI top 10	F top 10	MI top 3	F top 3	LASSO Features
QLattice	0.959	-	-	-	-	-
LASSO	0.959	-	-	-	-	-
Elasticnet	0.944	-	-	-	-	-
Random Forest	0.968	0.948	0.961	0.941	0.951	0.964
Gradient Boosting	0.975	0.959	0.95	0.946	0.916	0.963

Table 3. Benchmarks for Hepatocellular Carcinoma dataset

	All Features	MI top 10	F top 10	MI top 5	F top 5	LASSO Features
QLattice	0.633	-	-	-	-	-
LASSO	0.568	-	-	-	-	-
Elasticnet	0.611	-	-	-	-	-
Random Forest	0.604	0.628	0.558	0.594	0.541	0.604
Gradient Boosting	0.570	0.621	0.541	0.591	0.59	0.594

Table 4. Benchmarks for Breast Cancer dataset

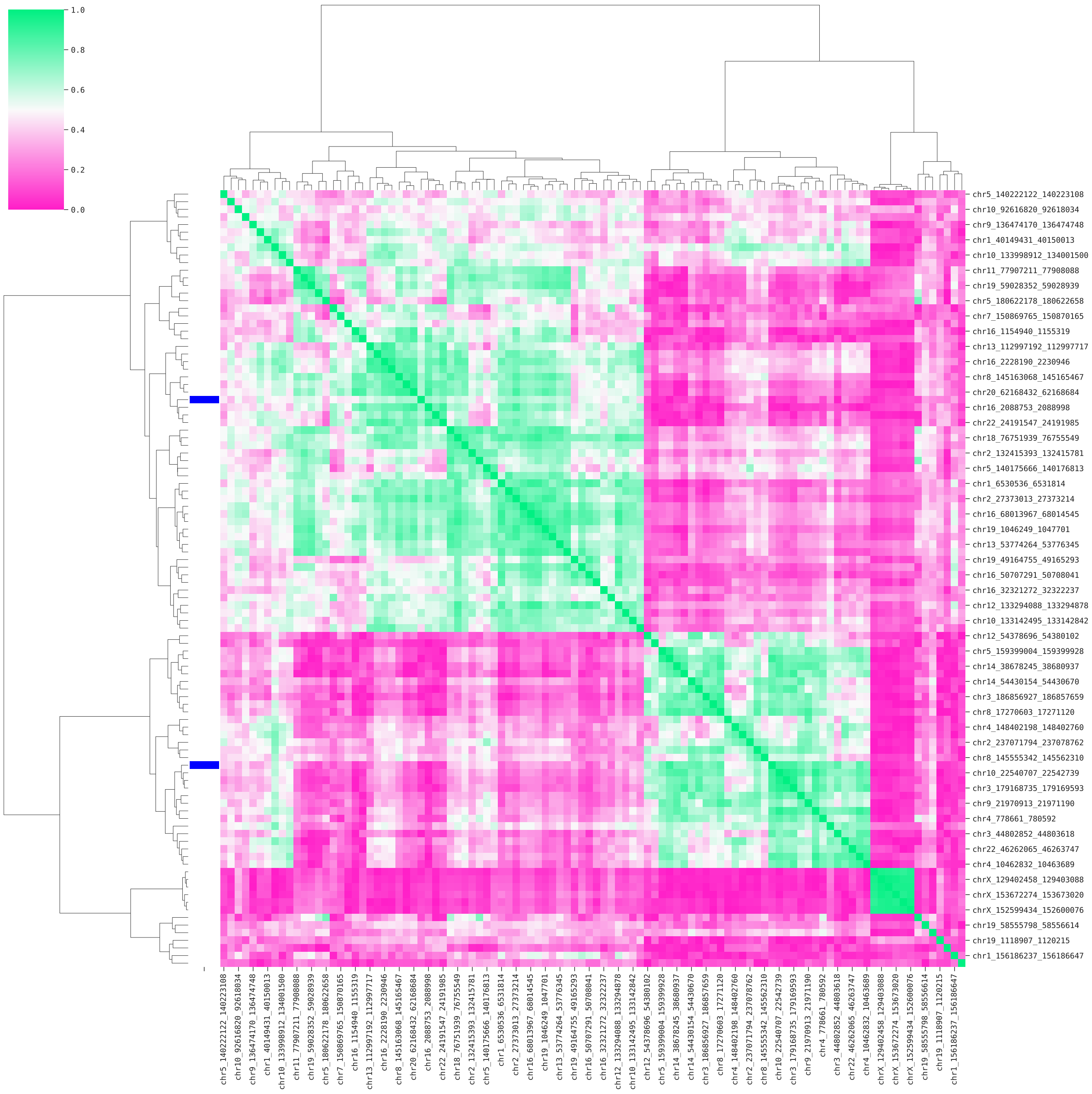

Fig. 1. HCC correlation heatmap for pairwise correlations (in absolute value) between a random subset of 100 features including the two model features (blue bars on the left). The heavy green coloring confirms extensive multi-collinearity. The plot clusters features based on similarity measured by the Pearson correlation coefficient followed by sorting. The two main groups of linear feature variance are displayed by the top branches in the dendrogram. Clustermap function from Seaborn Waskom [2021].

Figures

References

Cawley, G. C. and Talbot, N. L. C. (2010). On over-fitting in model selection and subsequent selection bias in performance evaluation. *Journal of Machine Learning Research*, **11**(70), 2079–2107.

Pedregosa, F., Varoquaux, G., Gramfort, A., Michel, V., Thirion, B., Grisel, O., Blondel, M., Prettenhofer, P., Weiss, R., Dubourg, V., Vanderplas, J., Passos, A., Cournapeau, D., Brucher, M., Perrot, M., and Duchesnay, E. (2011). Scikit-learn: Machine learning in Python. *Journal of Machine Learning Research*, **12**, 2825–2830.

Virtanen, P., Gommers, R., Oliphant, T. E., Haberland, M., Reddy, T., Cournapeau, D., Burovski, E., Peterson, P., Weckesser, W., Bright, J., van der Walt, S. J., Brett, M., Wilson, J., Millman, K. J., Mayorov, N., Nelson, A. R. J., Jones, E., Kern, R., Larson, E., Carey, C. J., Polat, İ., Feng, Y., Moore, E. W., VanderPlas, J., Laxalde, D., Perktold, J., Cimrman, R., Henriksen, I., Quintero, E. A., Harris, C. R., Archibald, A. M., Ribeiro, A. H., Pedregosa, F., van Mulbregt, P., and SciPy 1.0 Contributors (2020). SciPy 1.0: Fundamental Algorithms for Scientific Computing in Python. *Nature Methods*, **17**, 261–272.

Waskom, M. L. (2021). seaborn: statistical data visualization. *Journal of Open Source Software*, **6**(60), 3021.

Wilstrup, C. and Kasak, J. (2021). Symbolic regression outperforms other models for small data sets. *CoRR*, **abs/2103.15147**.
